# Pharmacological treatment of increased vascular risk and cognitive performance in middle-aged and old persons: six-year observational longitudinal study

**DOI:** 10.1186/s12883-020-01822-0

**Published:** 2020-06-12

**Authors:** Marlise E. A. van Eersel, Sipke T. Visser, Hanneke Joosten, Ron T. Gansevoort, Joris P. J. Slaets, Gerbrand J. Izaks

**Affiliations:** 1 University Center for Geriatric Medicine, University of Groningen, University Medical Center Groningen, AA41, PO Box 30.001, 9700 RB Groningen, The Netherlands; 2 Department of Pharmacy, PharmacoTherapy, -Epidemiology and -Economics (PTE2), University of Groningen, Groningen, the Netherlands; 3 Department of Internal Medicine, Maastricht University Medical Center, Maastricht, The Netherlands; 4 Department of Nephrology, University of Groningen, University Medical Center Groningen, Groningen, The Netherlands

**Keywords:** Cognitive performance, Treatment of increased vascular risk, Observational longitudinal analysis, Cardiovascular disease, Preventing cognitive impairment

## Abstract

**Background:**

Lowering vascular risk is associated with a decrease in the prevalence of cardiovascular disease and dementia. However, it is still unknown whether lowering of vascular risk with pharmacological treatment preserves cognitive performance in general. Therefore, we compared the change in cognitive performance in persons with and without treatment of vascular risk factors.

**Methods:**

In this longitudinal observational study, 256 persons (mean age, 58 years) were treated for increased vascular risk during a mean follow-up period of 5.5 years (treatment group), whereas 1678 persons (mean age, 50 years) did not receive treatment (control group). Cognitive performance was three times measured during follow-up using the Ruff Figural Fluency Test (RFFT) and Visual Association Test (VAT), and calculated as the average of standardized RFFT and VAT score per participant. Because treatment allocation was nonrandomized, additional analyses were performed in demographic and vascular risk-matched samples and adjusted for propensity scores.

**Results:**

In the treatment group, mean (SD) cognitive performance changed from − 0.30 (0.80) to − 0.23 (0.80) to 0.02 (0.87), and in control group, from 0.08 (0.77) to 0.24 (0.79) to 0.49 (0.74) at the first, second and third measurement, respectively (*p*_*trend*_ < 0.001). After adjustment for demographics and vascular risk, the change in cognitive performance during follow-up was not statistically significantly different between the treatment and control group: mean estimated difference, − 0.10 (95%CI − 0.21 to 0.01; *p* = 0.08). Similar results were found in matched samples and after adjustment for propensity score.

**Conclusion:**

Change in cognitive performance during follow-up was similar in treated and untreated persons. This suggests that lowering vascular risk preserves cognitive performance.

## Background

Worldwide, the prevalence of dementia is expected to reach 131.5 million persons in 2050 [1]. Because, up till now, no curative treatment is available, there is an increasing urge to prevent dementia in its earliest stages [1]. Cardiovascular diseases and dementia share similar pathogenetic processes, such as atherosclerosis, activated by common vascular risk factors like hypertension and hypercholesterolemia [2]. Therefore, it is generally assumed that treatment of vascular risk factors could be an effective strategy to preserve cognitive performance.

The effect of pharmacological treatment of vascular risk factors on cognitive performance was investigated in various randomized-controlled trials (RCTs) but overall, the results have been inconclusive. Only the Systolic Hypertension in Europe (Syst-Eur) trial and the Systolic Blood Pressure Intervention Trial (SPRINT-MIND) suggested a protective effect of antihypertensive treatment on dementia in contrast to other trials [3–6]. Furthermore, intensified treatment of diabetes mellitus or hypercholesterolemia had no effect on cognitive performance in RCTs like the ADVANCE study and the PROSPER trial [7, 8]. These negative findings may be explained by the fact that the intervention in these RCTs was focused on only one vascular risk factor. As this limitation was acknowledged in the Finnish Geriatric Intervention Study to Prevent Cognitive Impairment and Disability (FINGER), Prevention of Dementia by Intensive Vascular Care (preDIVA) study and Multidomain Alzheimer Preventive Trial (MAPT), these trials investigated the effect of a multidomain vascular intervention [9–11]. As described previously [12], in the FINGER trial, the multidomain vascular intervention was associated with better cognitive performance. However, the intervention in the FINGER trial also included cognitive training which could have influenced the positive findings considerably [9]. In the preDIVA trial, on the other hand, there was no effect of the multidomain vascular intervention on cognitive performance possibly due to the similar reduction in vascular risk in the intervention and control group [10]. Also, in the MAPT trial there was no association between cognitive function and multidomain intervention. However, this multidomain intervention included besides treatment of vascular risk factors also physical activity, cognitive training and nutritional advice [11]. Therefore, it is still unknown whether only pharmacological treatment of all vascular risk factors is positively associated with cognitive performance.

Nevertheless, considering observational data, a positive effect of pharmacological treatment on cognitive performance seems plausible. Recently, for example, we found that the change in cognitive performance was dependent on treatable vascular risk in both middle-aged and old persons during a mean follow-up period of 6 years [12]. Thus, in contrast to the trials described above [9–11], our findings suggested that pharmacological treatment of increased vascular risk could have an effect on cognitive performance. As the mean age of the study population in our study (54 years) was clearly lower than the average age of the study populations in the trials (69, 75, and 75 years, respectively) [9–12], a possible explanation for the negative results of the trials might be that pharmacological treatment was prescribed too late to preserve cognitive performance [13, 14]. Although our study had an observational design, we were able to explore the effect of pharmacological treatment on cognitive performance if it was started at younger age by extending the dataset of our previous study with detailed data on (cardiovascular) drug use [12].

The aim of this observational longitudinal study was to compare the change in cognitive performance of persons with and without pharmacological treatment of vascular risk factors over a follow-up period of nearly 6 years. The study included 1934 persons aged 35–82 years, who completed two to three measurements of cognitive performance and vascular risk.

## Methods

As this analysis is part of a longitudinal study, its methods partly overlap with those in other studies published by our group [12, 15].

### Study design

The study was part of the Prevention of REnal and Vascular ENd-stage Disease (PREVEND) study. As described previously [16, 17], the aim of the PREVEND study was to investigate prospectively the natural course of microalbuminuria and its association with renal and cardiovascular diseases in a cohort from the general population. The inclusion of participants in PREVEND study started in 1997–1998 (baseline). In brief, all 85,421 inhabitants of the city of Groningen, the Netherlands, aged 28–75 years were invited to participate in this study and to submit a first-morning-void urine sample. A total of 40,856 (48%) people responded. Participants were selected based on their urinary albumin excretion (UAE): 3395 with UAE < 10 mg/dl and 7768 with UAE > 10 mg/dl. A total of 8592 participants completed the baseline survey and were followed over time. The cognitive tests were introduced at the third survey of the PREVEND study (2003–2006). A total of 4135 participants completed the first measurement of cognitive performance and underwent repeated testing in the fourth survey (2006–2008) and/or fifth survey (2008–2012). Ultimately, 3601 participants completed two to three measurements of cognitive performance. Additionally, all surveys included also assessments of demographic, anthropometric and vascular risk factors, and measurements of haematological and biochemical parameters. Further details of the PREVEND study can be found in Mahmoodi et al. and Lambers Heersink et al. [16, 17]. A flow diagram of the participants, who completed measurements of cognitive performance at the third, fourth and fifth survey, can be found in van Eersel et al. [15].

As described previously [12], the PREVEND study was approved by the medical ethics committee (METc) of University Medical Center Groningen, Groningen, the Netherlands, and conducted in accordance with the guidelines of the Helsinki declaration. All participants gave written informed consent at baseline and agreed to follow-up over time. People who were not able to understand the invitation letter and submit a urine sample in the screening phase (due to cognitive impairment or other reasons) were considered incapable of giving informed consent and excluded. People who did not understand questions or instructions at follow-up were also considered incapable in regard to informed consent and consequently excluded from further participation.

### Allocation of treatment groups

Allocation was nonrandomized. Participants were allocated to the treatment group if they had pharmacological treatment of vascular risk factors for the first time ≤ 100 days before the first measurement of cognitive performance and continued treatment during follow-up. Participants were allocated to the control group if they did not have any pharmacological treatment of vascular risk factors during the whole study period. Treatment of vascular risk factors included pharmacological treatment of hypertension, hypercholesterolemia, diabetes mellitus and prevention of arterial thrombotic events. Pharmacological data were obtained from the IADB.nl prescription database. IADB contains prescriptions from 54 community pharmacies in the Netherlands and covers a population of 500,000 people. Both the age distribution and the prevalence of drugs used are comparable with that of the total Dutch population [18].

### Cognitive performance

As described previously [12], the cognitive performance was measured with a composite cognitive score of the Ruff Figural Fluency Test (RFFT) and the Visual Association Test (VAT). This composite score was calculated as follows (per participant and per measurement): the raw scores of the RFFT and the VAT were standardized to *z*-scores. Subsequently, the two resulting *z*-scores were averaged. The calculation of the *z*-scores was based on the mean and standard deviation of each test at the first measurement.

In general, the RFFT is used as a measure of executive function. However, it also yields important information about other cognitive abilities such as initiation, planning, divergent thinking and the ability to shift between different cognitive tasks. The lowest (worst) score is 0 points, the highest (best) score is 175 points [19, 20]. The RFFT is a sensitive measure and can be used to detect changes in cognitive performance across a wide age range [19, 21]. The VAT is a brief learning task and is commonly used to evaluate memory impairment including anterograde amnesia. The lowest (worst) score is 0 points, the highest (best) score is 12 points [22].

### Covariates

As described previously [12], data on age, gender and educational level were obtained from a questionnaire. In contrast to previous analyses, educational level was divided into two groups: low level (0–12 years of education) and high level (≥13 years of education) [23]. A history of cardiovascular events was defined as a prior cardiac, cerebrovascular or peripheral vascular event requiring hospitalization and was derived from the Dutch national registry of hospital discharge diagnoses during follow-up.

The vascular risk was measured by the Framingham Risk Score for Cardiovascular Disease (FRS-CD) [24]. As described previously [12], treatable vascular risk was calculated on the treatable components of the FRS-CD: diabetes mellitus (yes/no), current smoker status (yes/no), systolic blood pressure (mmHg), total cholesterol (mmol/l), HDL cholesterol (mmol/l) and use of blood pressure lowering drugs (yes/no) [24]. A detailed description of the measurements of the separate risk score components can be found in van Eersel et al. [12].

### Propensity score

A propensity score balances covariates in observational studies associated with the prescription of medication and is used to reduce bias by indication in non-randomized studies [25]. In this study, the estimated propensity score for treatment of vascular risk factors was calculated by a logistic regression model. The dependent variable was treatment of vascular risk factors (yes, no). The independent variables were age, gender, educational level, race, history of cardiovascular disease, family history of cardiovascular disease, body mass index, waist circumference, presence of diabetes mellitus, smoking, cholesterol, systolic blood pressure, diastolic blood pressure, presence of left ventricular hypertrophy, presence of albuminuria, use of alcohol, regular physical exercise, social situation, work situation, and net income per month (see Supplemental Table 1*)*. These independent variables were selected because in other studies, it was found that they are (potentially) associated with the prescription of treatment of vascular risk factors whereas they may also be associated with cognitive performance [26, 27]. Because the focus of the regression model was on optimal prediction, every initial variable was left in the model, regardless of the level of statistical significance of its coefficient. The R square of the full model was 0.37.

**Table 1:** Characteristics of the study population at the first measurement (baseline)

	All	Control	Treatment^a^	*P* value^b^
n (%)	1.934 (100)	1.678 (100)	256 (100)	N/A
Age (years), mean (SD)	51 (10)	50 (10)	58 (10)	< 0.001
Age groups, n (%)				< 0.001
35 to 44 years	542 (28)	520 (31)	22 (9)	
45 to 54 years	713 (37)	641 (38)	72 (28)	
55 to 64 years	449 (23)	357 (21)	92 (36)	
65 to 74 years	190 (10)	133 (8)	57 (22)	
≥ 75 years	40 (2)	27 (2)	13 (5)	
Gender, n (%)				< 0.001
Men	916 (47)	760 (45)	156 (61)	
Women	1018 (53)	918 (55)	100 (39)	
Educational level, n (%)				< 0.001
Low (≤12 years)	624 (32)	498 (30)	126 (49)	
High (≥13 years)	1310 (68)	1180 (70)	130 (51)	
Race, n (%)				
Western-European	1849 (96)	1604 (96)	245 (96)	0.62
Other	72 (4)	61 (4)	11 (4)	
Cardiovascular history^c^, n (%)	19 (1)	4 (< 1)	15 (6)	< 0.001
Treatable vascular risk (points)^d^, mean (SD)	1 (3)	1 (3)	4 (3)	< 0.001
Cognitive performance				
RFFT (points), mean (SD)	74 (26)	76 (25)	64 (25)	< 0.001
VAT (points), mean (SD)	10 (2)	10 (2)	9 (2)	< 0.001
Composite *z*-score^e^, mean (SD)	0.03 (0.78)	0.08 (0.77)	−0.30 (0.80)	< 0.001

Abbreviations: *RFFT* Ruff Figural Fluency Test, *VAT* Visual Association Test, *SD* Standard deviation, *N/A* Not applicable

^a^ Treatment group included persons who had treatment of vascular risk factors for the first time at the first measurement of cognitive function

^b^*P* values refer to comparisons between persons with and without treatment of vascular risk factors

^c^ All nineteen persons with a cardiovascular history had a cardiac event. There were no cerebrovascular of peripheral vascular event

^d^ Treatable vascular risk is based on the components of Framingham Risk Score for Cardiovascular Disease that are amenable to treatment and included diabetes mellitus, current smoker status, total cholesterol, HDL-cholesterol, systolic blood pressure and use of blood pressure lowering medication [24]

^e^ Cognitive performance was measured as a composite score of two tests (*z*-score): the Ruff Figural Fluency Test (RFFT) and the Visual Association Test (VAT) [20, 22]

### Matching

A matched subsample of participants with and without pharmacological treatment of increased vascular risk was created by one-to-one matching on age, gender, educational level and treatable vascular risk.

### Statistical analysis

Normally distributed data are presented as mean and standard deviation (SD), and skewed data are presented as median and interquartile range (IQR). Differences were tested by *t* test or, if appropriate, Mann-Whitney *U* test. Differences in proportion were tested by Chi-Square test. Trends across measurements were analyzed by ANOVA for normally distributed data and by the Kruskal-Wallis H test for skewed data.

The longitudinal association of cognitive performance with pharmacological treatment of increased vascular risk was investigated by linear multilevel analysis (linear mixed model analysis). Cognitive performance was the dependent variable. Pharmacological treatment of vascular risk factors (yes, no) was the independent variable. Participants were included in the analysis if they had completed both cognitive tests on at least two measurements. Consecutive measurement number (1,2,3) was the lowest level and participant the highest level. In this model, a significant main effect for treatment indicates an overall treatment effect over all three measurements. The interaction term treatment x consecutive measurement number was added to assess the treatment effect at the different measurements (1,2,3). First, adjustment was made for consecutive measurement number, age, interaction age x consecutive measurement number, educational level and treatable vascular risk. Second, adjustment was made for consecutive measurement number and propensity score. In all models, the continues variables were cognitive performance, age (years) and treatable vascular risk (points). Treatment of vascular risk factors (yes, no), consecutive measurement number (1,2,3) and educational level were categorical variables. The level of statistical significance was set at 0.05. All analyses were performed using IBM SPSS Statistics 22.0 (IBM, Armonk, NY).

In addition, as a consequence of its design, the participants of the PREVEND study had a somewhat higher prevalence of microalbuminuria than the general population (10% vs. 8%, respectively) [28]. Because this may influence data analyses, different statistical analyses of the PREVEND study were repeated in a subset of the PREVEND cohort, the Groningen Random Sample (*n* = 1651), which had a similar prevalence of microalbuminuria (8%) and other vascular risk factors as the general population as described in Lambers Heerspink et al. [17]. Essentially similar results were found if the analyses of cognitive performance with different vascular risk factors were repeated in the Groningen Random Sample compared to the analyses in the whole PREVEND cohort as described in Joosten et al. and van Eersel et al. [12, 29, 30].

## Results

Some data on the study population and the change in cognitive performance have been described previously [12, 15]. For convenience of the reader and to preserve continuity, the data are also presented in the following.

### Study population

**Fig. 1:**
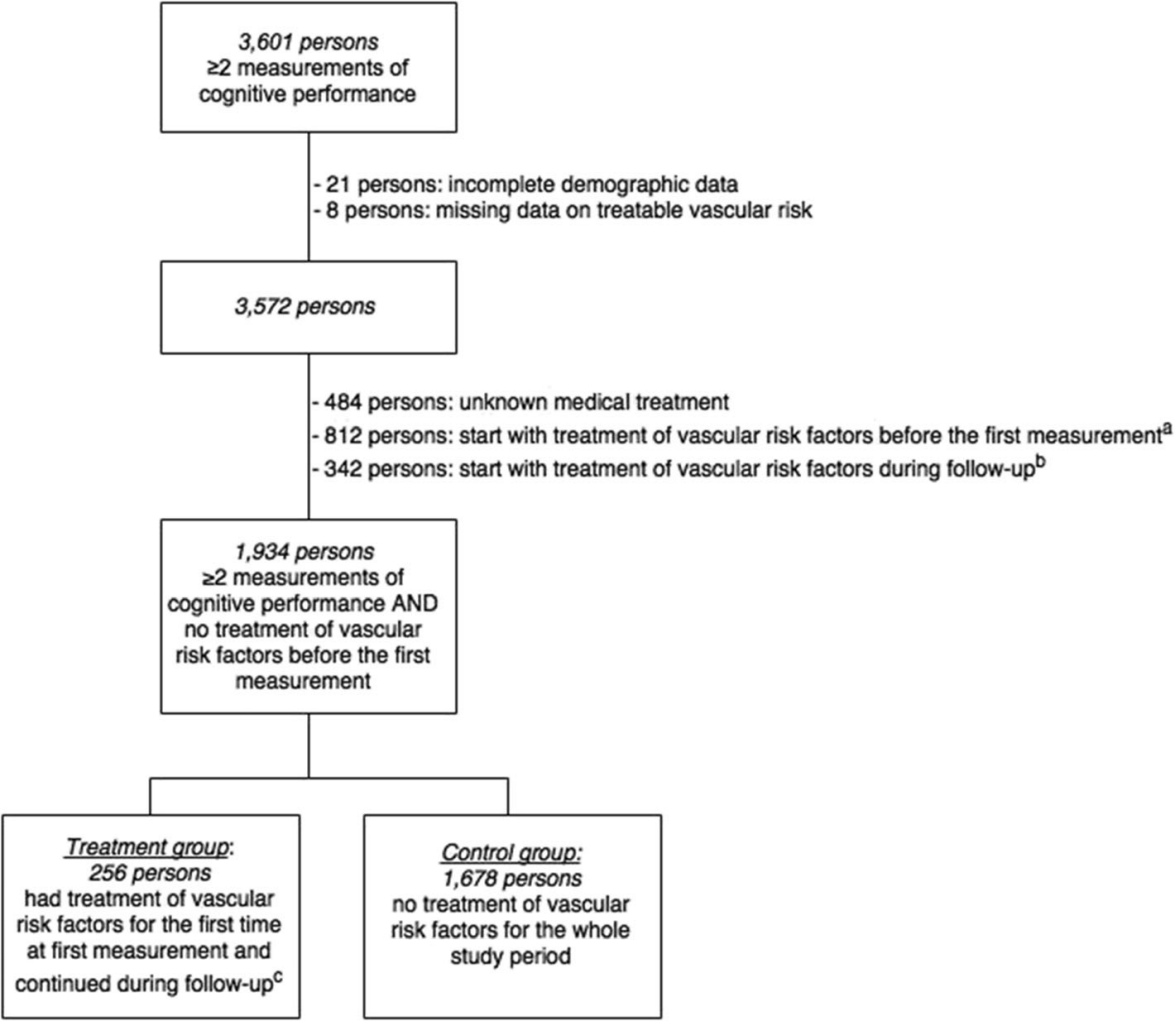
Flowchart of the selection of the study population. ^a^ Persons with treatment of vascular risk factors before the first measurement are persons with pharmacological treatment of hypertension, hypercholesterolemia, diabetes mellitus and prevention of arterial thrombotic events ≥100 days for the first measurement of cognitive performance. ^b^ Persons with treatment of vascular risk factors between the first and third measurement are persons who started with pharmacological treatment of hypertension, hypercholesterolemia, diabetes mellitus and prevention of arterial thrombotic events after the first measurement of cognitive performance. ^c^ Persons with treatment of vascular risk factors at first measurement are persons with for the first time pharmacological treatment of hypertension, hypercholesterolemia, diabetes mellitus and prevention of arterial thrombotic events ≤100 days before the first measurement of cognitive performance and continued treatment during follow-up

A total of 3601 persons completed the cognitive tests at multiple measurements: 2431 (68%) persons at three measurements and 1170 (32%) persons at two measurements. Of those, 21 persons (0.6%) were excluded because of incomplete demographic data and 8 persons (0.2%) because of missing data on treatable vascular risk. In addition, 484 (13%) persons were excluded because of missing data on pharmacological treatment and 1154 (32%) persons because of pharmacological treatment of vascular risk factors before the first measurement or treatment started during follow-up (Fig. 1). Finally, the total study population included 1934 persons. The mean age (SD) was 51 (10) years, 47% was men and 96% was of Western-European descent (Table 1).

Two hundred fifty-six persons (12%) had pharmacological treatment of vascular risk factors for the first time at the first measurement of cognitive performance and continued during follow-up. Persons in the treatment group were older and had a lower educational level compared to persons of the control group. The prevalence of cardiovascular history was higher in the treatment group. Also, persons of the treatment group had a higher treatable vascular risk than persons of the control group (Table 1). In addition, the treatable vascular risk of the treatment group did not change statistically significantly during follow-up despite pharmacological treatment of vascular risk factors (*p* = 0.41).

### Longitudinal change in cognitive performance

The mean (SD) duration of follow-up was 5.5 (0.7) years. As reported previously [15], the mean (SD) cognitive performance of the total study population increased between consecutive measurements from 0.03 (0.78) at the first measurement to 0.18 (0.81) at the second measurement and to 0.44 (0.77) at the third measurement (*p*_*trend*_ < 0.001).

**Fig. 2:**
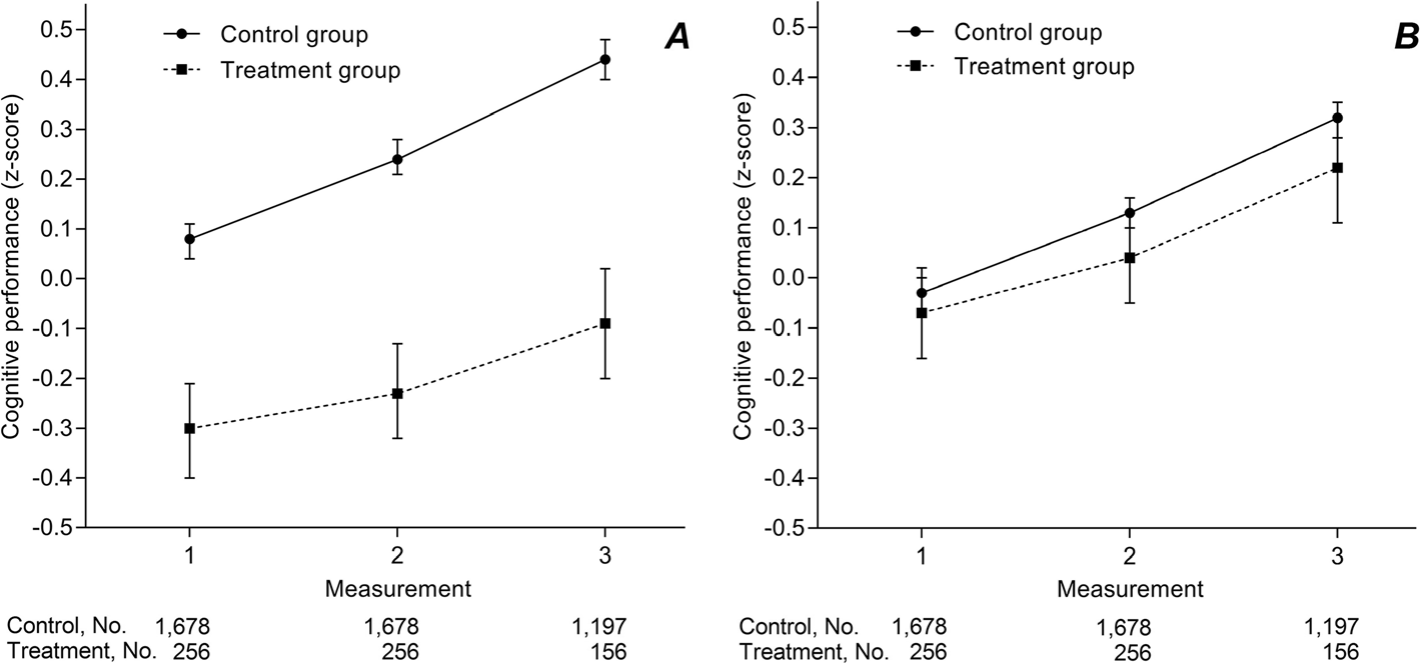
Mean cognitive performance during follow-up per control and treatment group. **a** unadjusted means. **b** covariate-adjusted estimated means from linear mixed models adjusted for age, educational level, interaction age x measurement and treatable vascular risk. Cognitive performance was measured as a composite score of two tests (*z*-score): the Ruff Figural Fluency Test (RFFT) and the Visual Association Test (VAT) [20, 22]. Bars represent 95% confidence intervals

The mean (SD) cognitive performance in the treatment group was lower than in the control group. In the treatment group, the mean (SD) cognitive performance changed from − 0.30 (0.80) to − 0.23 (0.80) to 0.02 (0.87) and in the control group, from 0.08 (0.77) to 0.24 (0.79) to 0.49 (0.74) at the first, second, and third measurement, respectively (*p*_*trend*_ < 0.001) (Fig. 2a).

### Adjustment for demographic factors and vascular risk

If the change in cognitive performance was adjusted for demographic factors and vascular risk, the difference in cognitive performance between the two groups was smaller (Fig. 2b). The covariate-adjusted linear mixed model analysis did not show a statistically significant overall treatment effect: the mean difference between the treatment and control group was − 0.07 (95%CI − 0.16 to 0.01; *p* = 0.08). Moreover, the estimated mean differences per measurement between the treatment and control group was only statistically significant after adjustment for demographic factors. However, it was not significant after additional adjustment for treatable vascular risk (Table 2).

**Table 2:** Difference in cognitive performance^a^ between treatment^b^ group and control group during follow-up: linear mixed model analyses

	Cognitive performance of treatment group as compared to control group (estimated mean difference)^c^								
	Model 1^d^			Model 2^e^			Model 3^f^		
	Mean	95%CI	*P* value	Mean	95%CI	*P* value	Mean	95%CI	*P* value
First measurement	−0.38	− 0.48 to − 0.28	< 0.001	− 0.10	− 0.20 to − 0.01	0.03	− 0.04	− 0.13 to 0.06	0.44
Second measurement	−0.47	− 0.57 to − 0.37	< 0.001	−0.15	− 0.24 to − 0.06	0.002	−0.09	− 0.18 to 0.01	0.08
Third measurement	−0.53	−0.65 to − 0.41	< 0.001	−0.16	− 0.27 to − 0.05	0.003	−0.10	− 0.21 to 0.01	0.08

Abbreviations: 95%CI, 95% confidence interval

^a^ Cognitive performance was measured as a composite score of two tests (*z*-score): the Ruff Figural Fluency Test (RFFT) and the Visual Association Test (VAT) [20, 22]

^b^ Treatment group included persons who had pharmacological treatment of vascular risk factors for the first time at the first measurement of cognitive function

^c^ Estimated mean difference was calculated as mean cognitive performance of treatment group minus control group

^d^ Model 1 is unadjusted; − 2*log likelihood: 10103.76

^e^ Model 2 is adjusted for age, educational level, interaction age x measurement; − 2*log likelihood: 9378.05

^f^ Model 3 is adjusted for age, educational level, interaction age x measurement, treatable vascular risk; − 2*log likelihood: 9348.58

### Adjustment for propensity score

For 1685 (87%) persons, a propensity score for pharmacological treatment of increased vascular risk could be calculated. If the change in cognitive performance was adjusted for propensity score, the covariate-adjusted linear mixed model analysis did not show a statistically significant overall treatment effect: the mean difference between treatment and control group was − 0.06 (95%CI − 0.18 to 0.06; *p* = 0.32). Moreover, at none of the three measurements, the estimated mean difference between the treatment and control group was not statistically significant (Table 3).

**Table 3:** Difference in cognitive performance^a^ between treatment^b^ group and control group during follow-up: linear mixed model analyses

	Cognitive performance of treatment group as compared to control group (estimated mean difference)^c^											
	Total study population (*n* = 1685)						Matched sample 1:1 (*n* = 478)^d^					
	Model 1^e^			Model 2^f^			Model 3^g^			Model 4^h^		
	Mean	95%CI	*P* value	Mean	95%CI	*P* value	Mean	95%CI	*P* value	Mean	95% CI	*P* value
First measurement	−0.35	− 0.46 to − 0.24	< 0.001	0.03	−0.10 to 0.16	0.68	−0.05	− 0.20 to 0.10	0.53	− 0.05	−0.17 to 0.08	0.49
Second measurement	−0.45	−0.56 to − 0.34	< 0.001	−0.07	− 0.21 to − 0.06	0.28	−0.07	− 0.22 to 0.08	0.36	− 0.07	−0.20 to 0.06	0.28
Third measurement	−0.51	−0.64 to − 0.39	< 0.001	−0.14	− 0.28 to 0.00	0.06	− 0.09	−0.26 to 0.07	0.27	−0.08	− 0.23 to 0.07	0.29

Abbreviations: *95%CI* 95% confidence interval

^a^ Cognitive performance was measured as a composite score of two tests (*z*-score): the Ruff Figural Fluency Test (RFFT) and the Visual Association Test (VAT) [20, 22]

^b^ Treatment group included persons who had pharmacological treatment of vascular risk factors for the first time at the first measurement of cognitive function

^c^ Estimated mean difference was calculated as mean cognitive performance of treatment group minus control group

^d^ The sample (*N* = 478) was matched on the following characteristics: age, gender, educational level and treatable vascular risk

^e^ Model 1 is unadjusted; − 2*log likelihood: 8851.36

^f^ Model 2 is adjusted for propensity score for treatment of vascular risk factors; − 2*log likelihood: 8752.82

^g^ Model 3 is unadjusted; − 2*log likelihood: 2521.53

^h^ Model 4 is adjusted for age, educational level, interaction age x measurement, treatable vascular risk; − 2*log likelihood: 2319.52

### Matched samples

Overall, 239 persons from the treatment group could be matched one-to-one to the control group. There were no statistically significant differences between the matched samples in age, gender, educational level or treatable vascular risk (*p* > 0.58). On average, the treatment sample had a slightly lower cognitive performance than the control sample at all measurements. In the treatment sample, the mean (SD) cognitive performance changed from − 0.27 (0.80) to − 0.20 (0.80) to 0.05 (0.86) and in the control sample, from − 0.23 (0.79) to − 0.13 (0.91) to 0.17 (0.79), at the first, second and third measurement, respectively (*p*_*trend*_ < 0.001). The overall treatment effect was not statistically significant in linear mixed model analysis: the mean difference between the matched treatment and control sample was − 0.07 (95%CI − 0.21 to 0.07; *p* = 0.31). Moreover, at none of the three measurements, the estimated mean difference between the matched samples was not statistically significant (Table 3).

## Discussion

In this large community-based observational study of middle-aged and old persons, the mean change in cognitive performance in the treatment and control group was similar despite the fact that at baseline, the treatment group was older, had a higher treatable vascular risk and a worse cognitive performance. This suggests that the treatment of vascular risk factors preserves cognitive performance.

Our findings supported the results of the Finnish Geriatric Intervention Study to Prevent Cognitive Impairment and Disability (FINGER) [9]. In this RCT, a multidomain intervention including treatment of vascular risk factors maintained cognitive performance in elderly people during a follow-up period of 2 years [9]. However, our study differs from the FINGER trial in several aspects. While the FINGER trial included a study population of elderly people with a high risk of dementia, our study included a sample from the general population comprising both middle-aged and old persons. Moreover, the duration of follow-up in the FINGER trial was 2 years, whereas in our study, follow-up was more than 5 years. Most importantly, the effect of pharmacological treatment of vascular risk factors per se on cognitive performance is unclear in the FINGER trial as their multidomain intervention also included other treatment methods such as diet, physical activity and cognitive training [9]. In contrast, our study only compared the change in cognitive performance between persons with and without pharmacological treatment of vascular risk factors. Therefore, it probably gives more insight in the effect of vascular treatment per se in the general population.

Similarly, our results supported the findings of the Multidomain Alzheimer Preventive Trial (MAPT) [11]. In this trial, a multidomain intervention including physical activity, cognitive training and nutritional advice did not reduce cognitive decline in frail elderly with memory complaints during a follow-up period of 3 years [11]. However, this trail had the same shortcomings as the FINGER trial. The MAPT trial included not only elderly with complaints of cognitive dysfunction, but it is also unclear whether the multidomain intervention included pharmacological treatment of increased vascular risk [11, 31]. Therefore, the effect of pharmacological treatment of vascular risk factors per se on cognitive performance is also in this trial unknown.

Our findings were also in line with the Prevention of Dementia by Intensive Vascular Care (preDIVA) trial of elderly people [10]. In this RCT, intensive treatment of vascular risk factors did not result in a reduced incidence of all-cause dementia in the treatment as compared to the control group [10]. This result could possibly be explained by the fact that a similar reduction in vascular risk was achieved in the treatment and control group of preDIVA. In addition, the primary outcome of preDIVA (all-cause dementia) might not be sensitive enough to detect a difference between the treatment and control group as dementia is usually diagnosed at a relatively late phase compared to the moment when the first cognitive changes occur [10]. In contrast, our study investigated the change in cognitive performance as outcome. Probably, this is a more sensitive measure that may find relatively small differences between the treatment and control group at an earlier stage.

### Limitations and strengths

Some limitations of this study have to be noted, as was also mentioned in our previous publication [12]. Most importantly, our study had an observational design whereas it is generally acknowledged that the estimated treatment effects in observational studies may be higher than the treatment effect that is found in subsequent RCTs evaluating the same intervention [32]. Although some argue that only RCTs could draw conclusions on the impact of vascular risk management [33], RCTs evaluating the effect of treatment of increased vascular risk on cognitive performance are also hindered by important methodological challenges [34]. First, such RCTs require large samples and long follow-up, especially in people aged < 70 years. Second, the importance of vascular risk management to prevent cardiovascular disease is undisputed. Therefore, withholding or withdrawing treatment in control subjects for a long period would be unethical [33, 34]. As a consequence, the feasibility of such RCTs can be questioned. For that reason, we think that large observational cohort studies comprising middle-aged and old persons may add valuable insights to what is known from recent RCTs. To lower the risk of indication bias [25], we used propensity scores and matching. In our study, these approaches yielded similar results.

Another limitation may be the measurement of cognitive performance with two cognitive tests which may not measure all cognitive domains. However, the RFFT provides information about diverse cognitive abilities such as initiation, planning, divergent reasoning, and the ability to switch between different tasks [19, 20]. Furthermore, the RFFT was combined with the VAT as a measure of memory [22].

Also, it should be observed that in our study, the composite cognitive test score increased across the measurements in our study which was probably due to a practice effect by the repeated exposure to the test [15]. Nonetheless, a practice effect is dependent on the capacity to learn and therefore, can be considered as the result of different cognitive abilities [35, 36]. Moreover, the association of cognitive performance in our study was adjusted for repeated measurements by entering consecutive measurement number as an independent variable into the regression models.

Finally, our study may be underpowered to detect a statistically significant effect of the pharmacological treatment of increased vascular risk. Although we acknowledge that a larger study might have yielded other results, it could still be questioned whether the effect of the treatment would be clinically relevant. The estimated difference in cognitive performance between the treatment and control group that we found in our study was 0.08–0.14 *z*-score (treatment *worse* than control). This corresponded to a difference of 2–4 points on the RFFT and 0.2–0.3 points on the VAT. At the same time, it is known that in the general population, the average decrease in RFFT score amounts to 4 points *per 5 years of age* [21]*.* Thus, a change of 2–4 points in the RFFT score corresponds to an age-related change that on average, develops over the course of 5 to 10 years. Similarly, the study of Lindeboom et al. described that there is at least a difference of 4 points in VAT score between normal subjects and subjects with dementia [22]. Therefore, in our opinion, for both tests, these differences are far below the threshold of clinical relevance. Even if the differences between the treatment and control group would be statistically significant in a (much) larger study, they would still lack a clinical impact.

Although our study had several limitations, there are also various strengths. Our study population was selected from a large community-based cohort and included many middle-aged and old persons in contrast to the FINGER, preDIVA and MAPT trials who selected only old persons with a high risk of cognitive impairment [9–11]. In addition, our study only investigated the association of pharmacological treatment of increased vascular risk with cognitive performance and did not include other types of intervention like cognitive training [9–11].

## Conclusion

In conclusion, in this large community-based study, the change in cognitive performance during a follow-up period of nearly 6 years was similar in the treatment and control group. This suggests that pharmacological treatment of increased vascular risk preserves cognitive performance.

## Supplementary information

**Additional file 1: Supplemental Table 1.** The independent variables that were included in logistic regression model to calculate the estimated propensity score for treatment of vascular risk factors.

## Data Availability

The datasets used and/or analysed during the current study are available from the corresponding author on reasonable request.

## Abbreviations

RFFT: Ruff Figural Fluency Test
VAT: Visual Association Test
RCT: Randomized-controlled trial
Syst-Eur: Systolic Hypertension in Europe
SPRINT-MIND: Systolic Blood Pressure Intervention Trial – Memory and Cognition in Decreased Hypertension
ADVANCE: Action in Diabetes and Vascular Disease: Preterax and Diamicron Modified Release Controlled Evaluation
PROSPER: PROspective Study of Pravastatin in the Elderly at Risk
FINGER: Finnish Geriatric Intervention Study to Prevent Cognitive Impairment and Disability
preDIVA: Prevention of Dementia by Intensive Vascular Care
MAPT: Multidomain Alzheimer Preventive Trial
PREVEND: Prevention of REnal and Vascular ENd-stage Disease
UAE: Urinary albumin excretion
IADB: InterAction Database
FRS-CD: Framingham Risk Score for Cardiovascular Disease

## References

[1] Alzheimer's Disease International. World Alzheimer report 2015: the global impact of dementia: an analysis of prevalence, incidence, cost and trends. London; 2015. p. 1–84. https://www.alz.co.uk/research/WorldAlzheimerReport2015.pdf. Accessed on May 13, 2020.

[2] Qiu C, Fratiglioni L (2015). A major role for cardiovascular burden in age-related cognitive decline. Nat Rev Cardiol.

[3] Forette F, Seux ML, Staessen JA, Thijs L, Babarskiene MR, Babeanu S (2002). The prevention of dementia with antihypertensive treatment: new evidence from the systolic hypertension in Europe (Syst-Eur) study. Arch Intern Med.

[4] The SPRINT MIND Investigators for the SPRINT Research Group (2019). Effect of intensive vs standard blood pressure control on probable dementia: a randomized clinical trial. JAMA..

[5] Ligthart SA, Moll van Charante EP, Van Gool WA, Richard E (2010). Treatment of cardiovascular risk factors to prevent cognitive decline and dementia: a systematic review. Vasc Health Risk Manag.

[6] Gorelick PB, Furie KL, Iadecola C, Smith EE, Waddy SP, Lloyd-Jones DM (2017). Defining optimal brain health in adults. A presidential advisory from the American Heart Association / American Stroke Association. Stroke.

[7] ADVANCE Collaborative Group (2008). Intensive blood glucose control and vascular outcomes in patients with type 2 diabetes. N Engl J Med.

[8] Shepherd J, Blauw GJ, Murphy MB, Bollen ELEM, Buckley BM, Cobbe SM (2002). Pravastatin in elderly individuals at risk of vascular disease (PROSPER): a randomised controlled trial. Lancet..

[9] Ngandu T, Lehtisalo J, Solomon A, Levalahti E, Ahtiluoto S, Antikainen R (2015). A 2 year multidomain intervention of diet, exercise, cognitive training, and vascular risk monitoring versus control to prevent cognitive decline in at-risk elderly people (FINGER): a randomised controlled trial. Lancet..

[10] Moll van Charante EP, Richard E, Eurelings LS, van Dalen JW, Ligthart SA, van Bussel EF (2016). Effectiveness of a 6-year multidomain vascular care intervention to prevent dementia (preDIVA): a cluster-randomised controlled trial. Lancet.

[11] Andrieu S, Guyonnet S, Coley N, Cantet C, Bonnefoy M, Bordes S (2017). Effect of long-term omega 3 polyunsaturated fatty acid supplementation with or without multidomain intervention on cognitive function in elderly adults with memory complaints (MAPT): a randomised, placebo-controlled trial. Lancet Neurol.

[12] van Eersel MEA, Joosten H, Gansevoort RT, Slaets JPJ, Izaks GJ (2019). Treatable vascular risk and cognitive performance in persons aged 35 years or older: longitudinal study of six years. J Prev Alzheimers Dis.

[13] Alzheimer’s Disease International. World Alzheimer report 2014: dementia and risk reduction: an analysis of protective and modifiable factors. London; 2014. p. 1–104. https://www.alz.co.uk/research/WorldAlzheimerReport2014.pdf. Accessed on May 13, 2020.

[14] Dorresteijn JA, Kaasenbrood L, Cook NR, van Kruijsdijk RC, van der Graaf Y, Visseren FL (2016). How to translate clinical trial results into gain in healthy life expectancy for individual patients. BMJ..

[15] van Eersel ME, Joosten H, Koerts J, Gansevoort RT, Slaets JP, Izaks GJ (2015). Longitudinal study of performance on the Ruff figural fluency test in persons aged 35 years or older. PLoS One.

[16] Mahmoodi BK, Gansevoort RT, Veeger NJ, Matthews AG, Navis G, Hillege HL (2009). Microalbuminuria and risk of venous thromboembolism. JAMA..

[17] Lambers Heerspink HJ, Brantsma AH, de Zeeuw D, Bakker SJ, de Jong PE, Gansevoort RT (2008). Albuminuria assessed from first-morning-void urine samples versus 24-hour urine collections as a predictor of cardiovascular morbidity and mortality. Am J Epidemiol.

[18] Visser ST, Schuiling-Veninga CC, Bos JH, de Jong-van den Berg LT, Postma MJ (2013). The population-based prescription database IADB.Nl: its development, usefulness in outcomes research and challenges. Expert Rev Pharmacoecon Outcomes Res.

[19] Ruff R, Light R, Evans R (1987). The Ruff figural fluency test: a normative study with adults. Dev Neuropsychol.

[20] Ruff R (1996). Ruff figural fluency test: professional manual.

[21] Izaks GJ, Joosten H, Koerts J, Gansevoort RT, Slaets JP (2011). Reference data for the Ruff figural fluency test stratified by age and educational level. PLoS One.

[22] Lindeboom J, Schmand B (2003). Visual association test. Manual.

[23] United Nations Educational, Scientific and Cultural Organization (2006). International standard classification of education ISCED 1997. Re-edition 2006.

[24] D'Agostino RBS, Vasan RS, Pencina MJ, Wolf PA, Cobain M, Massaro JM (2008). General cardiovascular risk profile for use in primary care: the Framingham heart study. Circulation..

[25] Rubin DB (1997). Estimating causal effects from large data sets using propensity scores. Ann Intern Med.

[26] Schilling C, Mortimer D, Dalziel K, Heeley E, Chalmers J, Clarke P (2016). Using classification and regression trees (CART) to identify prescribing thresholds for cardiovascular disease. Pharmacoeconomics..

[27] Mohammed MA, El Sayed C, Marshall T (2012). Patient and other factors influencing the prescribing of cardiovascular prevention therapy in the general practice setting with and without nurse assessment. Med Decis Making.

[28] De Jong PE, Hillege HL, Pinto-Sietsma SJ, de Zeeuw D (2003). Screening for microalbuminuria in the general population: a tool to detect subjects a risk for progressive renal failure in an early phase?. Nephrol Dial Transplant.

[29] Joosten H, Izaks GJ, Slaets JP, de Jong PE, Visser ST, Bilo HJG (2011). Association of cognitive function with albuminuria and eGFR in the general population. Clin J Am Soc Nephrol.

[30] Joosten H, Visser ST, van Eersel ME, Gansevoort RT, Bilo HJG, Slaets JP (2015). Statin use and cognitive function: population-based observational study with long term follow-up. PLoS One.

[31] Gillette Guyonnet S, Van Kan A, Andrieu S, Aquino JP, Arbus C, Becq JP (2008). Prevention of progression to dementia in the elderly: rationale and proposal for a health-promoting memory consultation (an IANA task force). J Nutr Health Aging.

[32] Hemkens LG, Contopoulos-Ioannidis DG, Ioannidis JP (2016). Agreement of treatment effects for mortality from routinely collected data and subsequent randomized trials: meta-epidemiological survey. BMJ..

[33] Richard F, Pasquier F (2012). Can the treatment of vascular risk factors slow cognitive decline in Alzheimer's disease patients?. J Alzheimers Dis.

[34] Richard E, Andrieu S, Solomon A, Mangialasche F, Ahtiluoto S, Moll van Charante EP (2012). Methodological challenges in designing dementia prevention trials - the European dementia prevention initiative (EDPI). J Neurol Sci.

[35] Bartels C, Wegrzyn M, Wiedl A, Ackermann V, Ehrenreich H (2010). Practice effects in healthy adults: a longitudinal study on frequent repetitive cognitive testing. BMC Neurosci.

[36] Calamia M, Markon K, Tranel D (2012). Scoring higher the second time around: meta-analyses of practice effects in neuropsychological assessment. Clin Neuropsychol.

