# Supplementary material for: Pharmacological treatment of increased vascular risk and cognitive performance in middle-aged and old persons: six-year observational longitudinal study
Source: BMC Neurol. 2020 Jun 12;20:242. doi: 10.1186/s12883-020-01822-0 (PMC7291556; doi:10.1186/s12883-020-01822-0)
Supplement: Supplementary file 1 — Additional file 1: Supplemental Table 1. The independent variables that were included in logistic regression model to calculate the estimated propensity score for treatment of vascular risk factors. [file 12883_2020_1822_MOESM1_ESM.docx]

**ONLINE SUPPLEMENTAL**

Pharmacological treatment of vascular risk factors and cognitive performance in middle-aged and old persons: Longitudinal study

Marlise E.A. van Eersel, MD^1,^*; Sipke T. Visser^2^; Hanneke Joosten, MD, PhD^3^; Ron T. Gansevoort, MD, PhD^4^; Joris P.J. Slaets, MD, PhD^1^; Gerbrand J. Izaks, MD, PhD^1^

^1^ University of Groningen, University Medical Center Groningen, University Center for Geriatric Medicine, Groningen, The Netherlands

^2^ University of Groningen, Department of Pharmacy, PharmacoTherapy, -Epidemiology and -Economics (PTE2), Groningen, the Netherlands

^3^ Department of Internal Medicine, Maastricht University Medical Center, Maastricht, The Netherlands

^4^ University of Groningen, University Medical Center Groningen, Department of Nephrology, Groningen, The Netherlands

**Supplemental Table 1.** The independent variables that were included in logistic regression model to calculate the estimated propensity score for treatment of vascular risk factors.

| Group | Variables | R square of total model |
| --- | --- | --- |
| Demographic | Age (years) | 0.11 |
| factors | Gender (men, women) |  |
|  | Educational level (low, high) |  |
|  | Race (Western-descent, other) |  |
|  | Social situation (live alone, live with partner without children, live with partner and children, live without partner and with children), |  |
|  | Work situation (job, unemployed, unable to work, retired) |  |
|  | Net income per month (<€1,200, €1,200-1,799, €1,800-2,199, €2,200-2,799, €2,800-3,799, €3,800-5,800, >€5,800) |  |
| Vascular risk | Current smoker status (yes, no) | 0.35 |
| factors | Presence of diabetes mellitus (yes, no) |  |
|  | Cholesterol (mmol/L) |  |
|  | Systolic blood pressure (mmHg) |  |
|  | Diastolic blood pressure (mmHg) |  |
|  | Body mass index (kg/m^2^) |  |
|  | Waist circumference (cm) |  |
|  | Use of alcohol (yes, no) |  |
|  | Regular physical exercise (yes, no) |  |
|  | Presence of left ventricular hypertrophy on ECG (yes, no) |  |
|  | Presence of albuminuria ≥30mg/24 hours (yes, no) |  |
| Medical history | Presence of history of cardiovascular disease (yes, no) | 0.37 |
|  | Presence of family history of cardiovascular disease (yes, no) |  |
